# A Practical Application for Quantitative Brain Fatigue Evaluation Based on Machine Learning and Ballistocardiogram

**DOI:** 10.3390/healthcare9111453

**Published:** 2021-10-27

**Authors:** Yanting Xu, Zhengyuan Yang, Gang Li, Jinghong Tian, Yonghua Jiang

**Affiliations:** 1 Key Laboratory of Intelligent Operation and Maintenance Technology and Equipment for Urban Rail Transit of Zhejiang Province, Jinhua 321004, China; xuyanting@zjnu.edu.cn (Y.X.); tianjh@zjnu.edu.cn (J.T.); yonghua_j82@zjnu.cn (Y.J.); 2 College of Engineering, Zhejiang Normal University, Jinhua 321004, China; 3 Key Laboratory for Biomedical Engineering of Ministry of Education, Department of Biomedical Engineering, Zhejiang University, Hangzhou 310007, China

**Keywords:** brain fatigue, mental health, ballistocardiogram (BCG), machine learning, fiber-optic sensor, heart rate variability (HRV)

## Abstract

Brain fatigue is often associated with inattention, mental retardation, prolonged reaction time, decreased work efficiency, increased error rate, and other problems. In addition to the accumulation of fatigue, brain fatigue has become one of the important factors that harm our mental health. Therefore, it is of great significance to explore the practical and accurate brain fatigue detection method, especially for quantitative brain fatigue evaluation. In this study, a biomedical signal of ballistocardiogram (BCG), which does not require direct contact with human body, was collected by optical fiber sensor cushion during the whole process of cognitive tasks for 20 subjects. The heart rate variability (HRV) was calculated based on BCG signal. Machine learning classification model was built based on random forest to quantify and recognize brain fatigue. The results showed that: Firstly, the heart rate obtained from BCG signal was consistent with the result displayed by the medical equipment, and the absolute difference was less than 3 beats/min, and the mean error is 1.30 ± 0.81 beats/min; secondly, the random forest classifier for brain fatigue evaluation based on HRV can effectively identify the state of brain fatigue, with an accuracy rate of 96.54%; finally, the correlation between HRV and the accuracy was analyzed, and the correlation coefficient was as high as 0.98, which indicates that the accuracy can be used as an indicator for quantitative brain fatigue evaluation during the whole task. The results suggested that the brain fatigue quantification evaluation method based on the optical fiber sensor cushion and machine learning can carry out real-time brain fatigue detection on the human brain without disturbance, reduce the risk of human accidents in human–machine interaction systems, and improve mental health among the office and driving personnel.

## 1. Introduction

Brain fatigue usually emerges when people maintain sustained concentration during long-term cognitive tasks, which can manifest as restlessness, low mood, inattention, slow thinking, prolonged reaction time, decreased work efficiency, and increased error rate and so on [1]. In addition to primitive fatigue accumulation caused by factors such as heavy pressure in life and work, and poor sleep quality [2], brain fatigue has become one of the biggest negative factors affecting public mental health [3]. Prolonged suffering fatigue can lead to some mental disorders, especially for chronic fatigue syndrome [4,5,6]. It is also an important risk factor in various man-machine systems with high safety requirements, such as air traffic control, manned aerospace, car/aircraft driving, etc. [7]. According to the China High-Speed website, the proportion of accidents caused by the brain fatigue of drivers accounted for more than 40% in big traffic accidents on highways, which poses a great threat to people’s lives and property. Brain fatigue can affect the information resources allocation of working memory and significantly decrease the efficiency of information transmission [8]. The higher the degree of brain fatigue, the greater its negative impact [9]. Therefore, a more accurate understanding of brain fatigue degree can help people adopt more rational rest strategies.

Brain fatigue detection methods can be summarized as subjective evaluation, psychological indicators, facial features, and biomedical signals, as shown in Table 1. Since subjective evaluation and psychological indicators require additional task experiments, the users must stop their current task to do another specific task to complete the evaluation, which lacks practicality in the actual task [10]. In some previous studies, facial features were used in driving fatigue detection [11,12], but the accuracy was unstable on account of the changes in internal and external environments during data collection, and the use of cameras cannot guarantee the users’ privacy. In recent years, brain fatigue detection methods based on electrophysiological signals (such as electroencephalogram (EEG), electrocardiogram (ECG), magnetoencephalogram (MEG), etc.) have been widely used for exploring the neural mechanisms and detection methods of brain fatigue [13], and have a high accuracy compared to other kind of methods [14]. However, these methods based on electrophysiological signals require additional contact electrodes on the brain scalp or human body surface during the tasks. The operation processes are cumbersome, which easily causes psychological pressure on the users, and are not practical [15].

Recently, ballistocardiogram (BCG) signals have drawn extensive interests from the investigators in the field of health monitoring [16]. BCG can be a non-electrode contact, non-binding, non-invasive monitoring technology, and has been widely used in biomedical engineering [17,18,19]. It is the description of the small displacements of the human body caused by heart activities [16]. The rhythm of BCG is consistent with ECG, and the measured heart rate extracted from BCG signal agrees well with the commercial physiologic device [20]. Wang et al. reported that the measured values of BCG have no statistically significant differences compared with that of ECG in the time domain, frequency domain, and non-linear indicators in a calm state [21], which shows that BCG signal is a reliable method to extract heart rate. Therefore, BCG can be used as an accurate and effective non-disturbing detection method to evaluate the brain fatigue liking the ECG signal [22,23,24,25].

In recent years, sensors have been widely used to detect BCG signals including two kinds of popular sensors, piezoelectric film sensors and optical fiber sensors. Both of them can perform interference-free measurement and obtain human heart rate information without burden [16,20]. ECG signal has been widely used for brain fatigue detection [23,24,25,26], but lacks the application of a BCG signal in brain fatigue evaluation. In this study, a fiber-optic sensor embedded in a cushion was used to collect human BCG signals on a chair. On the one hand, an experiment was conducted to validate the dependability of the fiber-optic sensor cushion compared with a medical standard ECG monitor using participant’s heart rate. On the other hand, a group of mental arithmetic math problems were designed and implemented for brain fatigue induction to study the brain fatigue quantitative evaluation method based on machine learning and BCG. Our study is a practical application attempt for brain fatigue evaluation, which has great differences from the previous works (electrophysiological signal related), in that our method does not need to directly contact the human body and can quantify the brain fatigue degree.

## 2. Participants and Methods

### 2.1. Participants and Data Collections

**Figure 1:**
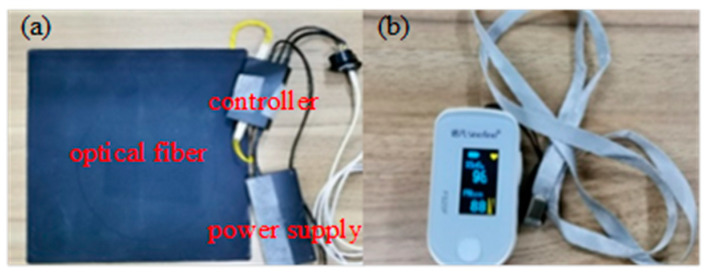
(**a**) Optical fiber sensor cushion embedded in the chair, (**b**) ECG monitor.

In this study, two groups of different volunteers participated in two experiments. Firstly, 20 healthy undergraduate students from Zhejiang Normal University were recruited. They were aged 19–23 years old and had no history of heart disease. Ten of them were male and 10 of them female. During the experiment, all subjects were asked to sit quietly for 5 min, until the subjects’ heart rates were relatively stable. Then a fiber-optic sensor cushion (shown in Figure 1a, provided by SHENGAO Technology Co., Ltd., Hangzhou, China) and the medical standard ECG monitor (shown in Figure 1b) were enabled to record 1 min of data at the same time. In addition, the fiber-optic sensor consists of three parts: optical fiber, controller, and power supply. According to the fiber-optic sensor manual, its measurement range is from 40–180 beats/min, and the measurement accuracy is ± 5 beats per min. Meanwhile, according to the medical standard ECG monitor, its measurement range is from 25–250 beats/min, and the measurement accuracy is ±2%.

Secondly, another 20 healthy participants (16 males and 4 females) from Zhejiang Normal University were included in the brain fatigue evaluation study. They were undergraduate students, graduate students, and young teachers. They were all right-handed, aged from 19–38 (23.3 ± 5.2). Their mean BMI was 20.7 ± 2.7 kg/m^2^. In order to control variables that may affect the results of the experiment, all recruited subjects were required to meet the following conditions: (a) No psychotropic drugs, no history of brain diseases, alcohol abuse, and no brain injuries; (b) No severe insomnia, no overnight stay, no drunkenness in the previous week before data acquisition; (c) No coffee, strong tea, and other refreshing internal drinks 2 h before the test. All subjects were required to complete a group of mental arithmetic math tasks, which was carried out for mental fatigue induction. The whole task includes 200 mental arithmetic math problems. Each math problem consists of adding two numbers between 60–99 and then multiplying by a number between 6–9. The 200 mental arithmetic math problems are all designed to have the same difficulty level. Each mental arithmetic math problem is required to be completed within 36 s, and the total experiment lasts 120 min. Mental arithmetic math task has often been used for inducing mental fatigue in previous works [27,28,29,30]. In our previous study, we used the same task and proved that mental fatigue did occur among healthy participants [31,32]. In another related mental fatigue study, Delliaux et al. analyzed fatigue, drowsiness, and anxiety at the same time, and found that fatigue and drowsiness emerged during long-duration switching task, and there was no statistical difference for anxiety before and after the task [33]. The task of mental arithmetic is more similar to the long-duration switching task and will not generate stress and anxiety during the task.

### 2.2. Computation of Heart Rate and HRV from BCG Signal

BCG signal is collected through the optical fiber sensor. The sampling rate of BCG signal is 10Hz. A band-pass filter (0.5–2.5 Hz) based on second-order Butterworth filter is applied on the BCG. Then heart rate is calculated based on the power spectrum. That is, the heart rate is equal to the spectrum peak multiplied by 60. The above algorithms are integrated in the controller of the optical fiber sensor. In other words, the sensor only outputs the heart rate.

**Figure 2:**
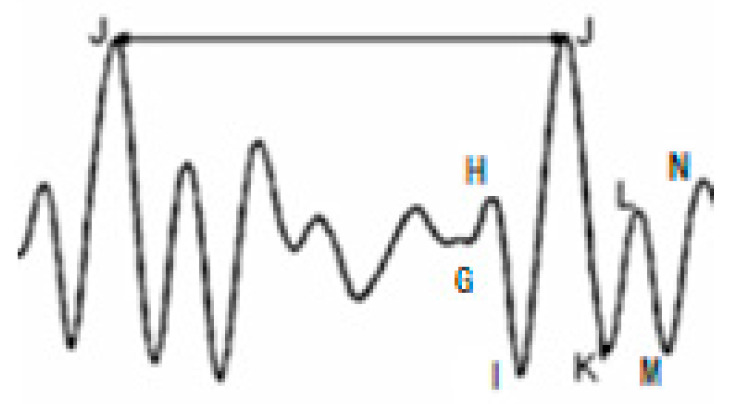
Typical BCG signal. Each BCG signal is characterized by several peaks and troughs reflecting specific events of the beating heart. The BCG of a complete cardiac cycle includes G, H, I, J, K, L, M, and N waves. J peak is the peak with the maximum amplitude produced when the heart beats at its maximum, I is the trough with the minimum amplitude before J peak, H is the peak before J peak, K and M are the troughs after J peak, and L and N are the peaks after J peak. The whole cardiac cycle can be divided into three parts: prosystolic (GH), systolic (IJK), and diastolic (LMN).

Heart rate variability (HRV) is a useful indicator to assess the changes of heart rate. As for healthy people, the body can quickly adapt to the changes in external and internal environments; the HRV value will be very high. When a person is ill or at fatigue state, the variability and complexity of the heartbeat activity will decrease, resulting in a decease of HRV. A typical BCG signal of a healthy person is shown in Figure 2. There are three main types of HRV analysis methods based on BCG signals [34], including time domain analysis (for example, the standard deviation of the sinus heartbeat J-J interval), frequency domain analysis, and nonlinear analysis (such as Lyapunov exponent, complexity, approximate entropy).

According to the description of HRV, this study proposes an indicator to characterize HRV based on frequency domain analysis. To be more specific, the ratio of reference heart rate to actual heart rate is put forward as the measurement of HRV. As shown in Equation (1), *h* is the actual heart rate value, and *h_ref_* is the reference heart rate. The average heart rate in first 10 min is used as the reference heart rate *h_ref_*. In order to expand the sensitivity of the characteristic value, the ratio is raised to the fourth power.

(1) $$V={\left(\frac{h_{ref}}{h}\right)}^{4}$$

### 2.3. Random Forest Model Construction

In this study, random forest is employed for brain fatigue detection [15], because it has high prediction accuracy, good tolerance for outliers and noise, and is not prone to overfitting. Random forest uses the integrated learning thinking to construct multiple independent decision trees based on randomly selecting sample information proposed by Breiman in 2001 [35], and establishes a classification model for the prediction target. When a new sample is input into random forest model, each decision tree will independently predict the classification result of the sample and vote for the result. Finally, the category with the most votes is determined based on the voted results of all decision trees. The steps of random forest model are as follows:

(a) Form the training set and testing set from the original data set;
(b) Randomly extract *d* features from the whole *D* features from the training samples (*d < D*);
(c) Construct random forest classification model with multiple decision trees and input the training set with the extracted *d* features into each decision tree;
(d) Enter testing set in the trained random forest model, each decision tree gets its own prediction result, then vote on the prediction result. It is judged that the category with the most votes is the final predicted result.

According to HRV values calculated by Equation (1), a random forest classifier is constructed to identify brain fatigue. The samples in the previous 10 min are used as the reference state (1 s can obtain one sample, that is, there are 12,000 samples for the reference state). Then every 10 min of samples is defined as a new state, which will be compared with the reference state by the random forest classifier. In addition, cross-validation strategy is applied for classification to make the accuracy more reliable. Eighty percent of the samples are randomly selected as the training set and the remaining 20% are selected as the validation set. The above calculations are repeated 20 times to obtain the final accuracy result, which is determined by averaging the accuracy of the 20 repetitions.

## 3. Results and Discussion

The test results of heart rate between fiber optic sensor and medical ECG monitor are shown in Table 2. The results showed that the absolute difference value of the heart rate measured by the optical fiber sensor and medical ECG monitor is not more than 3 beats/min, and the mean error is 1.30 ± 0.81 beats/min. In recent years, many studies have been carried out on the undisturbed measurement of heart rate, but they lack the application in brain fatigue detection. For example, Chen et al. designed a BCG monitoring system based on a seven-core fiber interferometer, and the mean difference between the standard monitored heart rate and their proposed sensor’s heart rate was 1.19 beats/min [20]. It can be seen that the fiber-optic sensor cushion used in this study can accurately and reliably detect and measure heart rate, which lays a foundation for HRV analysis and provides an accurate, reliable, and practical hardware foundation for quantitative brain fatigue evaluation.

**Figure 3:**
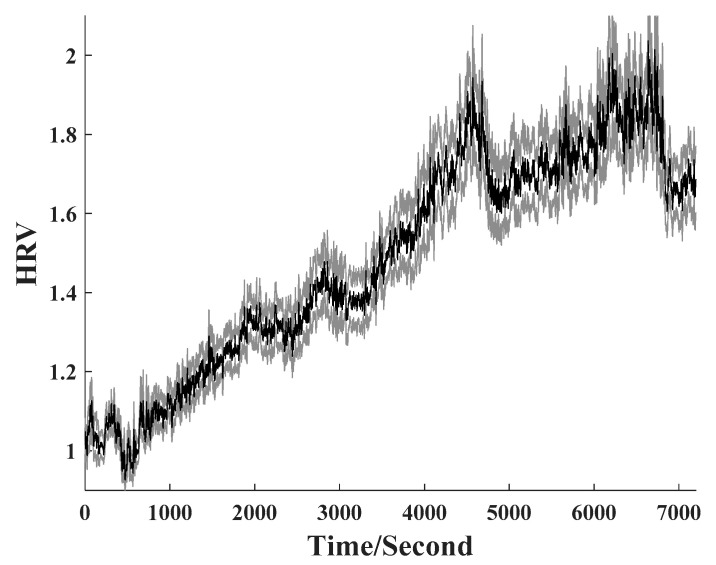
HRV changing trend along with the accumulation of mental arithmetic time. The shaded error bar represents the standard error of the mean HRV across 20 subjects.

HRV values during the whole test are shown in Figure 3. It is shown that HRV value presents an increasing trend with the accumulation of mental arithmetic time. Heart rate-related indicators have been widely used for mental fatigue evaluation. Laurent et al. reported that heart rate is decreased with the deepening of mental fatigue [36]. Delliaux et al. reported that heart rate significantly decreased and RR intervals, which is a commonly used indicator for HRV measurement, significantly increased with the increase of the task time [33]. As shown in Equation (1), the decrease of heart rate naturally resulted in the increase of HRV defined in this study. Our results are very consistent with previous studies [22,26,33], which suggested that the HRV index is an ideal method for evaluating brain fatigue. In addition, previous studies computed HRV from the sensors in direct contact with human body, which limited application scopes of these studies. Our study applied the fiber-optic sensor cushion in mental fatigue detection with high practicability.

The final classification accuracy results are shown in Table 3. The accuracy reached the highest amount of 96.5% when the mental arithmetic time lasted between 100 and 110 min. In some similar studies, the accuracy of fatigue classification is close to our study. Liu et al. obtained an accuracy of 92.7% with three non-hair-bearing EEG channels [15]. Laurent et al. reported an accuracy of 94 ± 2% for brain fatigue detection [36]. Ding et al. indicated that ECG signals have good discriminating power for mental workload detection with an accuracy of 96.4% [37]. It follows then that the brain fatigue evaluation method based on the optical fiber sensor cushion and the proposed HRV value in this study has high accuracy and reliability.

**Figure 4:**
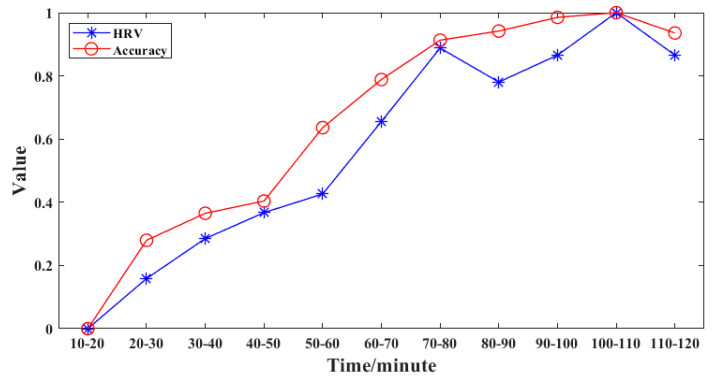
The correlation between the HRV and accuracy. As for the line of HRV shown in the figure, we averaged the values of HRV for every 10 min. Both HRV and accuracy are normalized between 0–1. So HRV can be compared with accuracy at the same size scale.

In addition, to our knowledge, this is the first time that the whole test was used for classification analysis. Previous studies always detected brain fatigue based on the data before and after the experiment [3], which ignored the dynamic evolution of brain fatigue. In this study, the whole test data were analyzed for brain fatigue evaluation. It can be seen from Table 3 that the accuracy presents an increasing trend and only has a small drop at the last 10 min, which is consistent with the HRV changing trend shown in Figure 3. On the basis of this interesting phenomenon, we further analyzed the correlation between HRV and the accuracy. The result is given in Figure 4. The correlation coefficient between HRV and accuracy was as high as 0.98, which indicates that the accuracy can be used as an indicator for quantitative brain fatigue evaluation during the whole task.

Our current study still has some limitations. On the one hand, only one task is used for mental fatigue induction. Our method should be verified in other kinds of tasks, especially in some practical applications. On the other hand, the goal of this study is to attempt to provide a practical application scheme for mental fatigue detection. The real-time algorithm has not been developed yet, and the time latency of the provided method cannot be measured. We will consider this issue in our future study.

## 4. Conclusions

In this study, we attempt to provide a strategy with application values for detecting and quantifying brain fatigue. Firstly, an undisturbed fiber-optic sensor embedded in a cushion was used to collect human BCG signals. An experiment was conducted to prove its reliability for heart rate extraction. The absolute error was less than 3 beats/min and the mean error was 1.30 ± 0.81 beats/min compared with a medical standard ECG monitor. The use of a fiber-optic sensor embedded in a cushion can ensure the practicality and reliability of our proposed strategy. Secondly, the HRV index extracted from human BCG signals was proposed for brain fatigue evaluation. Random forest classifier was applied on HRV index to quantify and recognize brain fatigue. The accuracy presented an increasing trend accumulation of mental arithmetic time and reached the highest accuracy of 96.5% when the task time lasted between 100 and 110 min. It can be seen that the proposed HRV index combined random forest classifier was an accurate and stable strategy for brain fatigue evaluation. In addition, the correlation between the proposed HRV index and the accuracy was analyzed, and the correlation coefficient was as high as 0.98, which indicated that the accuracy can be used as an indicator for quantitative brain fatigue evaluation during the whole task. To sum up, the method based on random forest and HRV index derived from the fiber-optic sensor can provide a practical, accurate, reliable, and stable strategy for quantitative brain fatigue evaluation.

## Figures and Tables

**Table 1:** Brain fatigue evaluation method.

Methods	Descriptions	Characters
Subjective evaluation [10]	Pearson Fatigue Scale, Stanford Sleepiness Scale, questionnaire survey, interview, etc.	Reliability and validity are low; they are often used as auxiliary research methods
Psychological indicators [14]	Sound and light response time, flash fusion frequency, etc.	Require additional design of mission experiments and equipment,; they are often used as auxiliary research methods
Facial features [11,12]	Percentage of eyelid closure, blink frequency, nodding frequency, yawn frequency, mouth state, etc.	No human contact, have strong practicability, but are easily affected by internal and external environments
Biomedical signals [13,14]	EEG, ECG, EMG, blood oxygen signal, respiratory signal, etc.	Often have touch with the human and have high accuracy and reliability

**Table 2:** Results of heart rate compared between fiber optic sensor and medical ECG monitor.

Subject Number	Heart Rate from Fiber Optic Sensor	Heart Rate from Medical Monitor	Absolute Error Value
1	76	78	2
2	82	81	1
3	80	83	3
4	76	75	1
5	85	86	1
6	83	85	2
7	80	80	0
8	81	82	1
9	77	79	2
10	94	93	1
11	81	83	2
12	78	78	0
13	83	85	2
14	77	78	1
15	75	75	0
16	78	79	1
17	96	96	0
18	87	89	2
19	78	81	3
20	78	79	1

**Table 3:** Accuracy results of HRV index with random forest classifier.

Time (min)	10–20	20–30	30–40	40–50	50–60	60–70	70–80	80–90	90–100	100–110	110–120
Accuracy (%)	72.30	79.08	81.15	82.09	87.72	91.41	94.43	95.13	96.18	96.54	94.98
